# Physicochemical properties and microstructure of corn flour–cellulose fiber extrudates

**DOI:** 10.1002/fsn3.2195

**Published:** 2021-03-16

**Authors:** Yilin Zhao, Chengbin Zhao, Xudong Tang, Jingyi Zhou, Hao Li, Hao Zhang, Jingsheng Liu

**Affiliations:** ^1^ College of Food Science and Engineering National Engineering Laboratory for Wheat and Corn Deep Processing Jilin Agricultural University Changchun China; ^2^ Department of Food Science Rutgers University New Brunswick NJ USA

**Keywords:** cellulose, corn flour, extrusion, microstructure, physicochemical properties

## Abstract

In this study, corn flour with 24% w/w moisture content was extruded, and cellulose at varied weight ratios was added in order to investigate its effect on the extrudate's physicochemical properties. Twin‐screw extrusion was divided into five temperature zones, and the screw temperature profile was 60℃, 120℃, 140℃, 120℃, and 110℃, respectively, and screw speed was 150 rpm. The cellulose content was 1%–15% w/w. Results showed that the addition of cellulose led to an increase in hardness, L^*^ and b^*^ of the extruded samples, and a decrease in degree of expansion, a^*^, thermal enthalpy of the sample paste. The sample paste exhibited a solid‐like characteristic. Microscopic morphology analysis showed that surface wrinkles of the sample increased with the increase of cellulose addition. The addition of cellulose can effectively increase the degree of puffing of corn flour–cellulose fiber extrudates.

## Graphical abstract

In this study, different ratios were selected to study the effect of cellulose on corn flour extrusion processing.

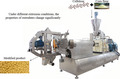

**Highlights**

1. Effect of cellulose additions at varied ratios on physicochemical properties of twin‐screw extruded cellulose–corn flour was studied.
2. Effect of twin‐screw extrusion process on cellulose–corn flour was explored, providing theoretical basis for corn starch and corn flour processing.

## 1 INTRODUCTION

Though people's living standards continues to improve, many health‐related disease arise. Obesity is one of the challenges and it seriously affects people's quality of life and health (Karri et al., 2019). To cope with this challenge, various weight‐control products are available on today's market, and additionally, consumers are paying more and more attentions to healthy diets. As a result, it is essential to ensure that functional characteristics and sensory characteristics are not destroyed during food processing, especially for natural or nature‐derived ingredient.

Cellulose is a polysaccharide with repeated units of glucose units. The human body mainly consumes plant products such as cereals, beans, vegetables, fruits, and seeds (Fuller et al., 2016). Other polyphenolic compounds (González‐Aguilar et al., 2017) and oligosaccharides (Macagnan et al., 2016) are also dietary fibers, further broadening the definition of dietary fiber. And some bioactive compounds are also important components of dietary fiber, which play an important role in human health (Zhu et al., 2018). Which can prevent or reduce intestinal diseases and reduce the risk of coronary heart disease and type 2 diabetes (Fuller et al., 2016). Total dietary fiber (TDF) can be categorized into two groups, that is, soluble dietary fiber (SDF) and insoluble dietary fiber (IDF) (Vasanthan et al., 2002). Important physicochemical properties of dietary fiber include solubility, viscosity, water content, swelling, and fermentability. Cellulose, hemicellulose, colloid, resistant starch, and nondigestible oligosaccharides are important dietary fibers. IDF usually leads to a decline in the sensory quality of the product. The fiber‐rich materials undergo modifications to their physicochemical and biochemical properties during extrusion‐cooking treatment to achieve the degradation of macromolecules, promoting the conversion of IDF to SDF in order to enhance the sensory quality. As the results of extrusion, the digestibility and water solubility of the released fiber fractions are greatly improved (Stojceska et al., 2009). However, the expanded volume of the soluble fiber is larger than that of the insoluble dietary fiber. During the extrusion process, the combination of starch and cellulose leads to a change in the degree of expansion, which has a great influence on the structure and physical and chemical properties of the starch (Altan et al., 2008). In addition, both soluble dietary fiber and insoluble dietary fiber have similar effects (Wu et al., 2015). Even though research on water‐soluble dietary fiber has been extensively studied, the research on cellulose has not yet been well studied.

The extrusion technology of food is roughly divided into two stages. The first stage was before the 1940s, focusing on the production of specific shaped foods such as enema, alfalfa, and macaroni. The second stage was after the 1940s. It is generally accepted that the extrusion process is a biological reaction at higher temperatures and has a shorter reflection time and can be modified by extrusion to obtain different intermediate or final products, such as extrusion of tissue proteins, modified starch, and modified fibers (Wiedmann & Strobel, 2004). From the second stage, extrusion technology has become an important food processing technology. As a new economical and practical food processing method, extrusion technology is widely used in food production because of its low cost, high efficiency, great versatility for diversified products, and low nutrient loss. Globally, research on extrusion puffing is becoming more and more in‐depth; instead of focusing on the research of extrusion parameters and product development, it is now mainly focusing on the process of extrusion on nutrients (protein (Emin et al., 2017), starch (Liu et al., 2017), fat (Engmann & Mackley, 2006),changes in dietary fiber (Monti et al., 2016), and interaction mechanisms. Fiber‐rich materials undergo significant physical and chemical changes during extrusion, and macromolecules degrade to promote the conversion of insoluble dietary fiber (IDF) to soluble dietary fiber (SDF) (Guo et al., 2018). Twin‐screw extrusion is a method to produce cellulose nanofibrils at a high solid content under continuous feeding (Trigui et al., 2020). Corn slang contains a higher content of cellulose, and corn flour used in the processing in this study is a whole corn flour, so the application of twin‐screw extrusion technology can also promote the conversion of IDF into SDF in corn slang, improve SDF content in the product, and achieve high efficiency. Taking advantage of the effect of reducing waste, Extrusion technology is a relatively complex food processing process used primarily to make ready‐to‐eat foods, including pasta, breakfast cereals, bread crumbs, biscuits, fried croutons, baby food, snacks, candies, chewing gum, and puffed plant proteins (Alam et al., 2016). Most staple food products can be obtained by extrusion, and extruded foods are favored by consumers because of their diverse shapes and raw materials (Nikmaram et al., 2015).

In the process of twin‐screw extrusion, when the material is extruded out of the die, the pressure suddenly decreases, the water quickly evaporates, and the material expands. The degree of expansion mainly depends on the composition of the material, the internal structure of the material when it leaves the die and the extrusion expansion parameters. Therefore, the degree of puffing can be used to determine the product quality of extruded puffed samples. Puffing degree is the main index to measure the quality of starch puffed products(Supat Chaiyakul et al., 2008). In this paper, the degree of puffing was used as the main index for the optimization of processing technology. This study provides a theoretical basis for starch extrusion food processing.

## 2 MATERIALS AND METHODS

### 2.1 Materials

Corn flour (CF) used for the study was provided by the laboratory (Changchun, Jilin), the corn variety is Xianyu335. Cellulose was provided by BEHRINGER (Beijing, China); the cellulose comes from corn and the cellulose purity is 97%. Corn flour was dried overnight at 45℃ and placed at 25℃ room temperature for use. Corn flour with cellulose addition amount of 1% to 15% was separately disposed.

### 2.2 Twin–screw extrusion process optimization

Single factor test was conducted to determine effects of water addition, extrusion temperature, and screw speed on puffing degree of the extruded puffed samples.

Effect of water addition: extrusion temperature was set at 140℃, screw speed was 150 r/min. Based on our previous research, we selected different water additions 20%, 22%, 24%, 26%, 28%, and 30% w/w (set to Group 1—Group 6), respectively, using a twin‐screw extruder. Extrusion test of corn flour was carried out to investigate effect of water addition on degree of expansion of the sample.
Effect of extrusion temperature: 25% w/w of moisture addition was used, screw speed was 150r/min. We selected different extrusion temperatures of 125℃, 130℃, 135℃, 140℃, 145℃, and 150℃ (set to Group 1—Group 6), respectively, using a twin‐screw extruder for corn. Extrusion test of powder was carried out to investigate effect of extrusion temperature on puffing degree of the sample.
Effect of screw speed: 25% w/w of moisture addition was used, extrusion temperature was set at 140℃. We selected different screw speeds, 130r/min, 140r/min, 150r/min, 160r/min, 170r/min, and 180r/min (set to Group 1—Group 6), respectively. Twin‐screw extruder was used to carry out extrusion test of corn flour, and effect of screw speed on puffing degree of the sample was also investigated.

### 2.3 Extrusion sample processing

Samples were processed in a laboratory twin‐screw FMHE 36–24 Co‐extruding Extruder (Fu ma C H, Hunan, CHN). The barrel diameter and L/D ratio were 36mm and 24D, respectively. A die head with a diameter of 4mm was used.. The temperature from the material entering the twin‐screw was as follows 60℃, 120℃, 140℃, 120℃, and 110℃, screw speed was 150 rpm, and feed rate was at 250 g/min. The corn flour (7.8% w/w moisture) was fed directly to the twin‐screw extruder, and the amount of water added was 24%, w/w and the puffed extruded samples extruded puffed sample was cut by a four‐leaf blade at a speed of 600 rpm. Random samples were taken from the extruded samples for subsequent experiments. Specific mechanical energy (SME; J/g) was calculated using the following (Kuoa et al., 2019):

SME=2πωτ0/M.

where ω is the screw speed (rpm), τ_0_ is the corrected torque (N· m), and M is the feed rate (g/min).

### 2.4 Viscosity

Viscosity of the sample powder was determined using a rapid viscosity meter (RVA‐TecMasterTM). 3 g sample was added to 25 ml of water. Heating and cooling cycles were carried out by the machine's own program, Standard 1. The procedure was to raise temperature to 50 ℃, and to 95 ℃ in 222 s, and then maintain the temperature for 150s, and lastly, the sample was cooled to 50 ℃ at the same rate for 120 s. The initial 10s was 960 rpm and then maintained at 160 rpm. Samples were repeated in triplicate in order to determine their gelatinization temperature, peak viscosity, final viscosity, disintegration value, rebound value, and lowest viscosity (Atwijukire et al., 2019).

### 2.5 Hardness

Hardness is defined as the maximum value (N) of the force required to break the sample, and the experiment is repeated in triplicate. In this experiment, P0.5 probe was mounted using TPA (Stable Micro System, UK) to determine hardness of the sample.

### 2.6 Degree of expansion

Five sections were randomly selected from nine gradient addition samples to measure degree of expansion. Diameter of the sample was measured with a vernier caliper, and degree of expansion (SEI: Sectional Expansion Index) was calculated by ratio of diameter of the sample to die opening. SEI = extruded sample diameter (mm)/die mouth diameter (mm).

### 2.7 Color value

Color of the sample was measured by a CM‐5 color difference meter (Konica‐Minolta, JPN), and each group was subjected to five parallel experiments to measure L^*^ a^*^ b^*^ value (L, a, b, respectively, representing chromaticity value of object color, that is, the color Color space coordinates, any color has its corresponding unique coordinate value).

### 2.8 Fourier transform infrared (FTIR) spectroscopy

Based on the method of Zhao (Zhao et al., 2017), infrared spectrum of the extruded sample was measured by VERTEX 70 FTIR spectrometer (Bruker, GER). Specifically, the sample was dried in an oven at 105℃ to remove internal free water. Weighed 1 mg of the sample into a mortar, added 150 mg KBr, grinded it under infrared light and mixed well, then put the mixed powder into a mold for tableting, and performed infrared spectrum scanning of the sample by Fourier infrared spectrometer. Scanning wavelength range was set at 400–4000 cm^‐1^ and resolution was 4 cm^‐1^.

### 2.9 Thermal characteristics

Thermal curve was measured by TA Q‐2000 Differential Scanning Calorimeter (TA Instruments, US). 2.5 mg of the sample was weighed, and then, 7.5 μL of water was added and evenly spread the sample on the bottom of the crucible to ensure that the crucible was free of residual moisture. Quality of the sample is lossless. Seal with a lid and press the crucible with a pressure applicator. Allowed to equilibrate enough and then put into TA Q‐2000 differential scanning calorimeter; nitrogen flow rate was 50ml/min, heating rate was set at 10℃/min, measuring range was between 50 and 90℃. DSC curve was calculated by computer program record and compared the analysis.

### 2.10 Microstructure

The sample was placed on sample holder using conductive paste, and gold was continuously sprayed at 10 Pa for 1 min. Finally, the sample was transferred to a scanning electron microscope, and microscopic morphology analysis was performed at a voltage of 5 kV.

### 2.11 Statistical analysis

The test was repeated 3 times and the average was taken as experimental result. Drawing was performed using Origin 8.5 software, and SPSS 19.0 software was used to process test data and significance analysis.

## 3 RESULTS AND DISCUSSION

### 3.1 Single factor experiment on optimization of corn flour extrusion process

**FIGURE 1:**
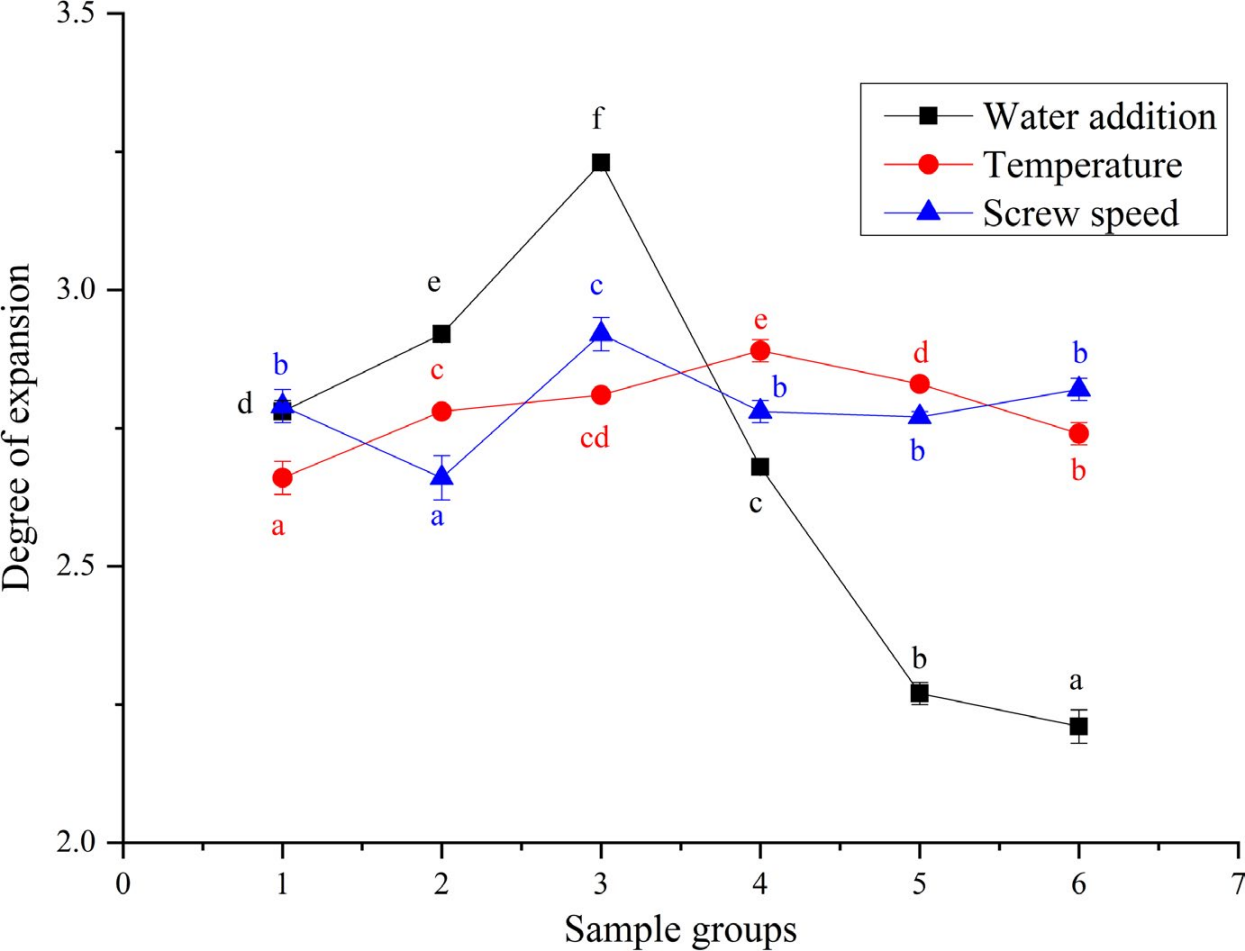
Effect of Different conditions on SEI on Extruded Samples. a‐f Means in the same group with different letters were significantly different (*p* < .05) from each other. (The same color represents the same group.)

Extrusion tests were carried out with water addition, extrusion temperature, and screw speed as single factor. Taking degree of expansion as an indicator, the results are shown in Figure 1.

It could be seen from Figure 1 that, with the increase of amount of water added during the extrusion process, decrease in degree of expansion. This was because when amount of water was increased, moisture content of the material in the sleeve was too high, and the material in the sleeve was diluted by water. Therefore, shearing force and friction in the sleeve were weakened, and residence time was shortened. As the result, when the material was extruded through die port, water vapor could not be completely evaporated, which hindered the generation of pores inside the extruded puffed samples, resulting in a decrease in degree of puffing. When lower amount of water was added, water molecules could not be completely combined with the material, and water absorption was uneven, which hindered formation of molten state of the material in the sleeve, resulting in poor overall fluidity of the material. Therefore, expansion of samples could not be well during extrusion, which led to a decrease in degree of expansion.

Additionally, it could be seen from Figure 1 that, as temperature increased during the extrusion process, degree of expansion of the extruded sample first increased and then decreased. This was because when extrusion temperature was low, the material was incompletely melted, viscosity was low, fluidity was good, and at the same time, water evaporated slowly when passing through the die, resulting in a decrease in degree of expansion. With the increase of temperature, water molecules in the sleeve were active, which promoted penetration of water molecules into the spatial structure of the material macromolecules. Under action of high pressure and shear force, macromolecular particles degraded, which was conducive to expansion of materials.

When extrusion temperature continued to rise and, exceeded 140℃, degree of expansion of the extruded samples showed a downward trend. This was because when temperature was high, water evaporated too quickly, and material moisture was lost. More, when the bulk was not fully expanded, the large amount of moisture would affect degree of expansion of the extruded samples, so that the material denatured at high temperature, easily became scorched inside the sleeve and formed a hard block, affecting its fluidity, resulting in its puffing. In severe cases, it is also easy to cause overload of the machine load.

It could be seen from Figure 1 that as screw rotation speed increased during the extrusion process, degree of expansion of the extruded sample first increased and then decreased. This was because when screw speed was low, the screw fed the material slowly, and there was no sufficient shearing force on the material. When the material passed through the die, there was not enough pressure to affect puffing degree of the extruded samples. As rotating speed of twin‐screw increased, shearing force of the screw increased gradually. Under the increasing shearing force, molecular space structure in the material increased, and water molecules could penetrate into the material. Molecular particle gap was beneficial to degradation of macromolecular particles in the extrusion process. At the same time, appropriate screw speed would also agitate, so that the further mixing of moisture and material was uniform, and structure and internal pores of the extruded samples were more uniform. When twin‐screw speed was higher, residence time of the material in the sleeve was shortened. Although higher rotation speed could provide sufficient shearing force and pressure, it also caused macromolecular substance in the material to be extruded out of the die without being completely broken and degraded in the sleeve, resulting in a decrease in degree of expansion. In summary, we choose the following extrusion conditions: screw speed 150 rpm, feed rate 250 g·min^‐1^, temperature zones, respectively, 60, 120, 140, 120, and 110 (℃), water addition 24%, and SME 187 J·g^‐1^.

### 3.2 Rapid viscosity analysis (RVA)

Pasting properties of starch are of particular importance in food industry because they determine the most appropriate utility of starches by the industry (Atwijukire et al., 2019).

As amount of cellulose added increased, peak viscosity, setback, and final viscosity were decreased. Since temperature during the gelatinization process was low, effect of temperature on changes of cellulose property could be neglected, and after cellulose was added to corn flour, cellulose acted only as the filler in the starch gelatinization process (Zhang et al., 2016). During the process of increasing temperature, starch molecules swelled to obtain a peak viscosity after washing completely. When cellulose was present, cellulose would affect mutual contact of starch molecules, resulting in a decrease in peak viscosity of starch. When starch molecules continued to absorb water and completely rupture, lowest viscosity was observed, and then, amylopectin and amylose molecules were intertwined, gelatinization ends, and the final viscosity was reached. In the process, cellulose molecules continued to act as fillers and hindered the intertwining of starch molecules without directly participating in changes of starch molecules, thus causing a decrease in retrogradation value and final viscosity.

It could be seen from Table 1 that peak viscosity, the lowest viscosity, and retrogradation value of corn flour were negatively correlated with amount of cellulose added. Since cellulose was insoluble in water, it had little binding effect with water and did not compete with starch molecules. Decreased viscosity may be due to a combined effect of insolubility of cellulose fibers in water and high water binding activity of cellulose. Moisture which caused starch granules to expand and gelatinize was increased, resulting in a decrease in viscosity. Cellulose also hindered formation of network complexes between starch–starch, thereby reducing gelatinization viscosity.

**TABLE 1:** Results of RVA

Cellulose addition/(wt%)	Pasting temperature/℃	Peak Viscosity/RVU	Trough viscosity/RVU	Final Viscosity/RVU	Breakdown value/RVU	Setback Value/RVU
0%	72.15 ± 0.63^a^	2,589.50 ± 16.06^a^	1842.5 ± 10.1^a^	3,683.00 ± 2.83^a^	747.00 ± 14.04^a^	1,840.50 ± 10.21^a^
1%	72.60 ± 1.31^ab^	2,423.5 ± 19.8^b^	1764.50 ± 17.07^ab^	3,514.50 ± 17.47^b^	659.00 ± 12.73^b^	1,750.00 ± 9.60^ab^
3%	73.075 ± 0.600^ab^	2,299.50 ± 11.61^b^	1675.00 ± 15.55^b^	3,326.50 ± 14.54^c^	624.50 ± 9.15^b^	1651.50 ± 10.01^bc^
5%	72.95 ± 0.64^ab^	2055.50 ± 9.19^c^	1503.50 ± 17.68^c^	3,035.00 ± 19.39^d^	552.00 ± 6.88^c^	1531.50 ± 7.08^d^
7%	73.025 ± 0.610^ab^	2018.50 ± 13.03^c^	1527.50 ± 10.20^c^	2,976.00 ± 15.29^d^	491.00 ± 2.82^c^	1,448.50 ± 4.75^d^
9%	73.05 ± 0.56^ab^	1709.50 ± 15.96^e^	1,312.00 ± 10.71^d^	2,614.50 ± 14.65^f^	397.50 ± 4.75	1,302.50 ± 5.02^ef^
11%	72.575 ± 0.030^ab^	1851.50 ± 13.03^d^	1,356.50 ± 11.51^d^	2,766.50 ± 12.81^e^	495.00 ± 8.48^c^	1,410.00 ± 11.21^de^
13%	73.475 ± 0.030^ab^	1663.00 ± 13.43^e^	1,258.50 ± 12.73^ed^	2,499.50 ± 15.26^f^	404.50 ± 0.71^d^	1,241.00 ± 2.53^g^
15%	74.275 ± 1.100^b^	1,497.50 ± 20.50^f^	1,172.00 ± 19.80^e^	2,259.50 ± 16.01^f^	325.50 ± 0.71^e^	1,087.50 ± 2.89^h^

Note

a‐h Means in the same columns with different letters were significantly different (*p* <.05) from each other.

### 3.3 Effect of amount of cellulose added on hardness of the extruded samples

Effect of addition of cellulose on hardness of the extruded samples was studied by a physical property meter. Results are shown in Table 2. Amount of cellulose added had a significant effect on changes in hardness of the extruded samples (*p* <.05). Hardness increased from 5.11g to 25.73g, with an increase of 403.52%. When amount of cellulose added was 5%‐11% w/w, difference in hardness of the extruded samples was not significant (*p* >.05), but hardness of the samples with other cellulose additions was significantly different (*p* <.05).

**TABLE 2:** Effect of Cellulose Addition on Hardness of Extruded Samples

Cellulose addition (wt%)	Hardness (*N*)
0%	5.11 ± 1.12^a^
1%	9.49 ± 1.48^b^
3%	16.40 ± 1.98^d^
5%	14.73 ± 1.62^cd^
7%	15.40 ± 3.75^cd^
9%	12.50 ± 1.69^bc^
11%	14.73 ± 1.56^cd^
13%	20.02 ± 1.52^e^
15%	25.73 ± 1.46^f^

Note

Different letters in the same columns (a, b, c…) represent significant differences (*p* <.05).

Changes of hardness of the extruded samples after addition of cellulose might be due to the increase of screw shearing effect caused by addition of cellulose, which led to aggregation of damage of the material melt, resulting in generation of pores. Cellulose as a filler could not be integrated into internal pores during extrusion, resulting in a denser structure and an increase in hardness.

### 3.4 Effect of amount of cellulose added on degree of expansion of the extruded samples

**FIGURE 2:**
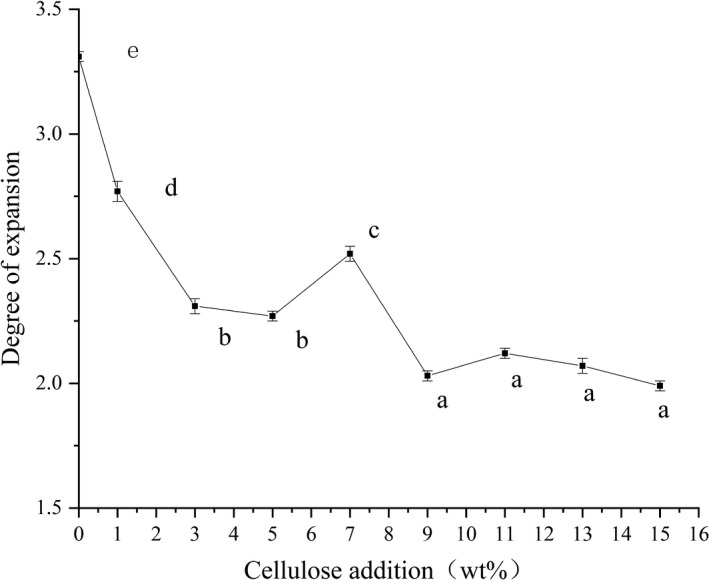
Effect of Cellulose Addition on SEI of Extruded Samples. a‐e Means in the same group with different letters were significantly different (*p* < .05) from each other

Degree of expansion of the extruded samples with different amounts of cellulose added was shown in Figure 2. Amount of cellulose added had a significant effect on changes in degree of expansion of the extruded samples (*p* <.05). When amount of cellulose added was 15% w/w, degree of puffing of the extruded samples was 1.99, which was 40.24% lower than that of the unfilled cellulose samples. Further, it was found that when amount of cellulose added was 9 to 15% w/w, difference in degree of expansion was not remarkable compared with other samples in which cellulose was added (*p* >.05).

Although solubility of cellulose might be improved, cellulose was still a water‐insoluble polysaccharide, and most of the cellulose was still dispersed as filler around starch molecules. During extrusion process of the sample, volume expansion first occurred when the material left the die, mainly because during the extrusion process, when the material left the die mouth, the cross‐sectional area was larger than the outlet due to its elastic recovery. An expansion effect was produced, which was called die swell. With the increasing amount of cellulose added, viscosity of the melt inside the sleeve increased, and thixotropy was weakened. When screw shearing action was received, damage was severe, resulting in a decrease in degree of expansion. Second, because of addition of cellulose, raw material and cellulose were mixed, melted, and sheared in the sleeve, and at the moment when the material was extruded into the die, pressure difference between the front and the back was too large, resulting in rapid evaporation of free water in the material. Intermolecular interaction between cellulose and starch was small, which caused pores generated by water vapor to be easily broken, which hindered further expansion of the sample. This was consistent with conclusion of hardness measurement.

### 3.5 Effect of amount of cellulose added on color value of the extruded samples

**FIGURE 3:**
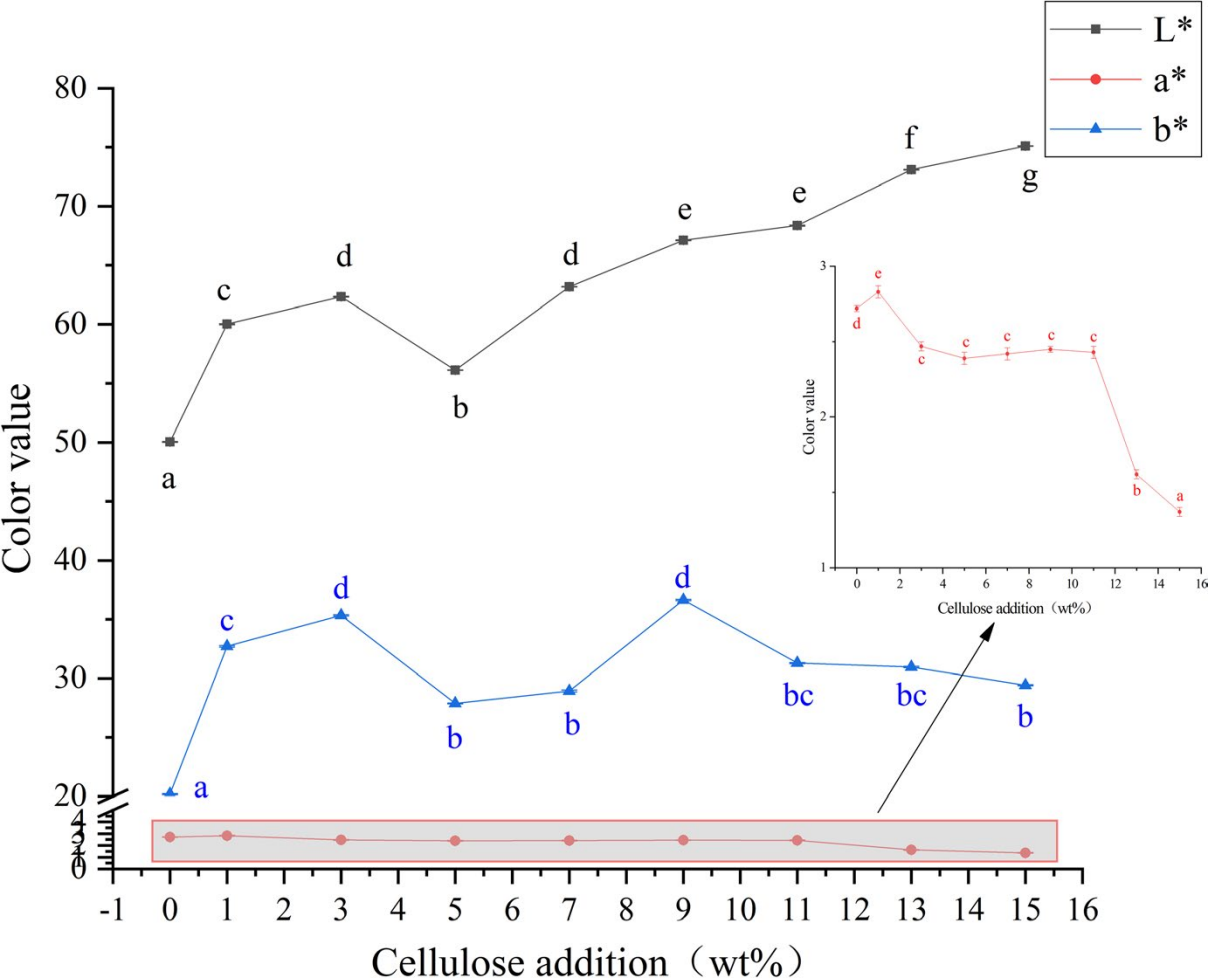
Effect of Cellulose Addition on Color Value of Extruded Samples. a‐e Means in the same group with different letters were significantly different (*p* < .05) from each other

Color value results of the corn powder extruded samples with different cellulose additions are shown in Figure 3. It could be seen from this figure that effect of addition of cellulose on L^*^, a^*^, and b^*^ of the extruded samples was significant (*p* <.05). When amount of cellulose added was 15%, L^*^ value was increased from 50.05 to 75.10, with an increase of 50.05%, compared to the sample without addition of cellulose. The a^*^ value was decreased from 2.72 to 1.37, with a decrease of 49.63%. When amount of cellulose added was 3%‐11%, difference in a^*^ value change was not significant compared with other cellulose addition samples (*p* >.05).

There were two main reasons for the increase in L^*^ value. Firstly, cellulose had a higher brightness; therefore, the addition of cellulose caused an increase in brightness of the extruded samples. Another reason for changes in brightness of the samples was due to Maillard reaction during the extrusion process, which resulted in a caramel or tan substance during the extrusion process, causing a decrease in brightness. The simultaneous effect of these two effects caused changes of L^*^, a^*^, and b^*^ value of the samples after extrusion.

### 3.6 Infrared spectrum analysis results of the extruded samples with different cellulose additions

Position of different absorption peaks in Fourier infrared spectrum corresponds to a specific functional group and can be used to analyze functional groups or chemical bonds in the samples (Xiang et al., 2018). Fourier transform infrared spectroscopy (FTIR) is used to study chemical structure, and peak position ranges from 3000–2800 cm^‐1^, 1760–1745 cm^‐1^, 1640–1620 cm^‐1^, 1400–1380 cm^‐1^, 1300–1000 cm^‐1^, and 970–840 cm^‐1^, respectively. C‐H stretching vibration, esterified C = O bond, COO‐ asymmetric stretching vibration, C‐H bending vibration and symmetric stretching vibration, C = O stretching vibration and ring stretching vibration. 2,365 cm^‐1^ is the background of CO_2_, and 2179–2000 cm^‐1^ is the stretching vibration of C≡C (Xiang et al., 2018).

**FIGURE 4:**
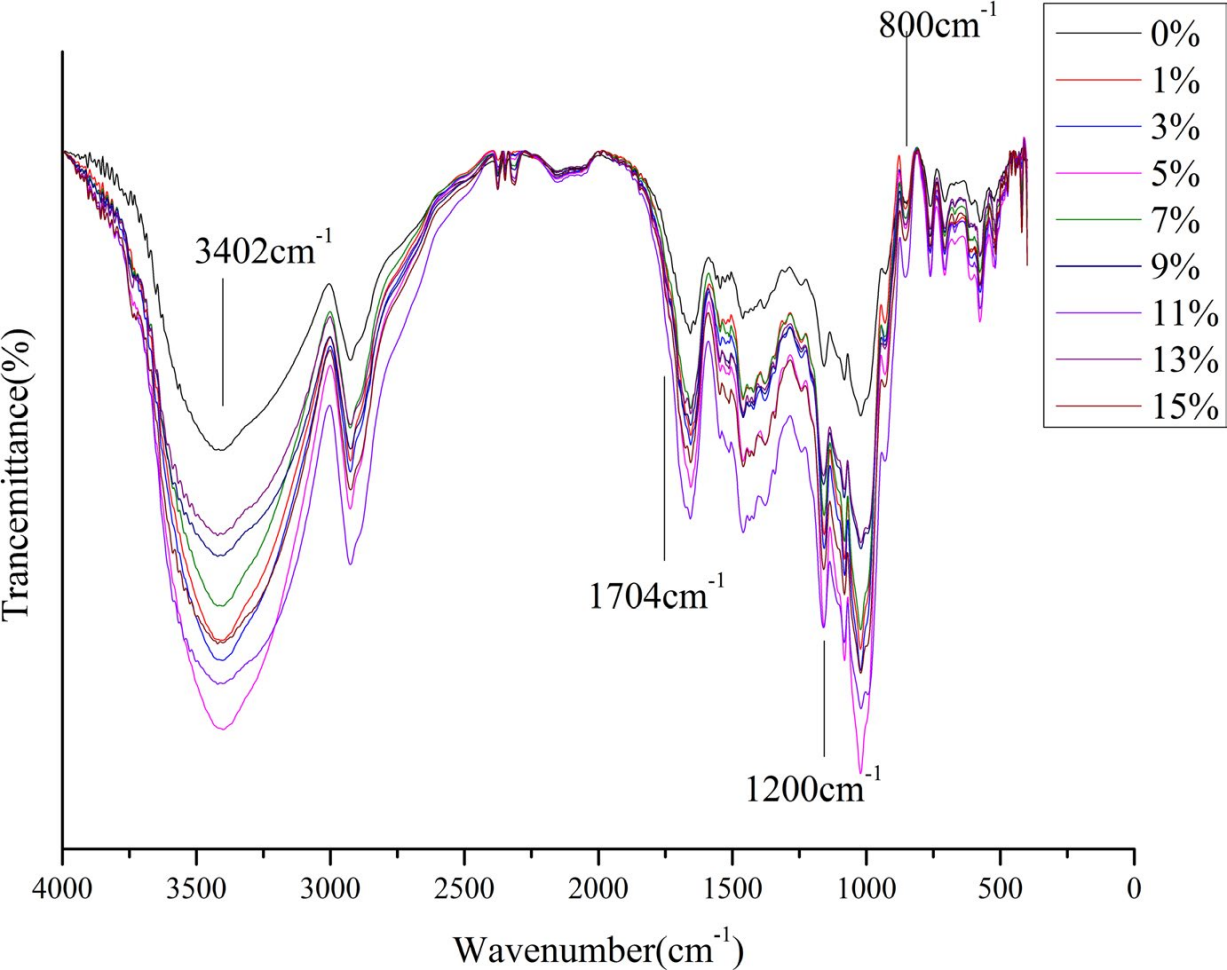
FTIR Spectroscopy Scanning of Extruded Cellulose–corn Flour (percentages denote percent of cellulose added)

Infrared spectrum scanning results of the corn flour extruded samples with different cellulose additions were shown in Figure 4. Characteristic peaks in the spectrum were similar, because starch and fiber were both carbohydrates and had similar chemical structures. Studies showed that outer structure of starch granules consisted of amylopectin and a large number of polymer linear segments. The interior of starch was mainly composed of gel crystals, and intensity of absorption peak in the infrared spectrum of starch particles in the range of 1200–800 cm^‐1^ could reflect molecular composition and short‐range order structure of starch.

No peak was observed at 1,740 cm^‐1^, resulting from destruction of C = O bond of cellulose acetate groups in cellulose by extrusion. Peak at 1532 cm^‐1^ gradually decreased with the amount of cellulose added, probably due to an increase in amount of cellulose added resulting in a decrease in protein and lipid in the original powder.

At vibration of the double bond of 1600–1500 cm^‐1^, the original powder had a distinct peak, and peak at the extruded sample was reduced, indicating that extrusion might destroy chemical structure of starch. 1651–1633 cm^‐1^ was δ‐OH bending vibration of water, representing bound water present in the sample. There were two distinct peaks at 1,080 cm^‐1^ and 1,022 cm^‐1^. The former was C‐O stretching vibration of the secondary alcohol hydroxyl group, and the latter peak was C‐O stretching vibration of the primary alcohol hydroxyl group.

930 cm^‐1^, 853 cm^‐1^, and 763 cm^‐1^ were characteristic absorption peaks of starch sugar ring, and characteristic peak of the extruded samples was weaker than that of the original powder.

### 3.7 Determination of thermal deuterium value of the extruded samples with different cellulose additions

**FIGURE 5:**
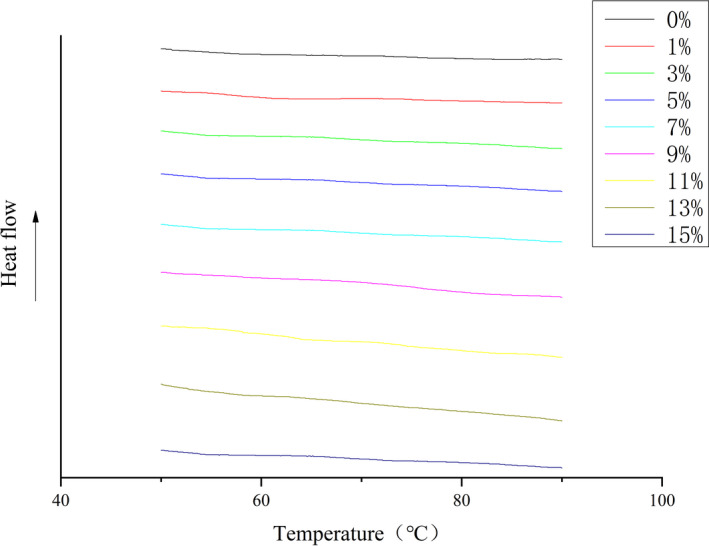
Cellulose–corn Extrusion Flour DSC Curve (percentages denote percent of cellulose added)

DSC scanning of different cellulose‐added extruded powders was carried out. Results are shown in Figure 5. Thermal enthalpy value of the extruded corn flour was substantially zero, indicating that starch gelatinization in corn flour was complete during the extrusion process, and substantially no ungelatinized starch granules were present.

Results of thermal characteristics of unextruded corn flour with different cellulose additions are shown in Table 3. As amount of cellulose increased, thermal enthalpy of cellulose–corn flour gradually decreased. When amount of cellulose was 15%, compared with the sample free of cellulose, enthalpy was reduced by 40.59%. Addition of cellulose would affect gelatinization process of corn flour. Firstly, in the cellulose–corn flour system, addition of cellulose reduced content of gelatinizable starch in corn flour in the unit gelatinization system to some extent, reducing starch concentration, which resulted in a decrease in gelatinization heat enthalpy and an increase in gelatinization temperature. After cellulose and corn flour were mixed, a partitioning effect on network structure in the starch gelatinization system was observed, resulting in increased gelatinization temperature and reduced thermal enthalpy, and secondly, due to hydrophilicity of cellulose, it had water swell ability. Addition of cellulose affected water activity in the cellulose–corn flour system, reduced water available during the gelatinization process, and hindered operation of water molecules, which caused gelatinization temperature to decrease. Combination of these two effects led to a reduction in thermal enthalpy.

**TABLE 3:** Cellulose–corn Extrusion Flour Thermal Properties

Cellulose addition (wt%)	Starting temperature(°C)	Peak temperature(°C)	Termination temperature(°C)	△H (J/g)
0%	58.31 ± 0.78^a^	72.71 ± 1.76^c^	88.19 ± 0.87^d^	7.08 ± 0.25^e^
1%	58.93 ± 1.82^ab^	70.50 ± 1.29^abc^	86.68 ± 0.96^cd^	6.46 ± 0.16^cd^
3%	60.34 ± 0.47^ab^	68.37 ± 0.55^a^	86.55 ± 1.14^cd^	6.09 ± 0.37^cd^
5%	60.42 ± 2.02^ab^	70.00 ± 0.34^ab^	85.36 ± 1.56^bc^	5.86 ± 0.26^bcd^
7%	60.95 ± 1.95^ab^	68.02 ± 0.92^a^	85.35 ± 0.34^bc^	5.56 ± 0.14^bcd^
9%	61.44 ± 0.53^ab^	69.80 ± 0.19^ab^	84.84 ± 0.96^bc^	5.19 ± 0.49^abc^
11%	61.66 ± 0.79^ab^	70.64 ± 0.33^abc^	84.59 ± 0.62^bc^	5.12 ± 0.37^abc^
13%	61.66 ± 1.75^ab^	71.06 ± 1.52^bc^	84.04 ± 0.57^b^	4.79 ± 1.05^ab^
15%	62.61 ± 1.56^b^	70.62 ± 0.16^abc^	81.55 ± 0.39^a^	4.21 ± 0.09^a^

Note

Different letters in the same columns (a, b, c…) represent significant differences (*p* <.05).

### 3.8 Scanning of microscopic morphology of the extruded samples with different cellulose additions

**FIGURE 6:**
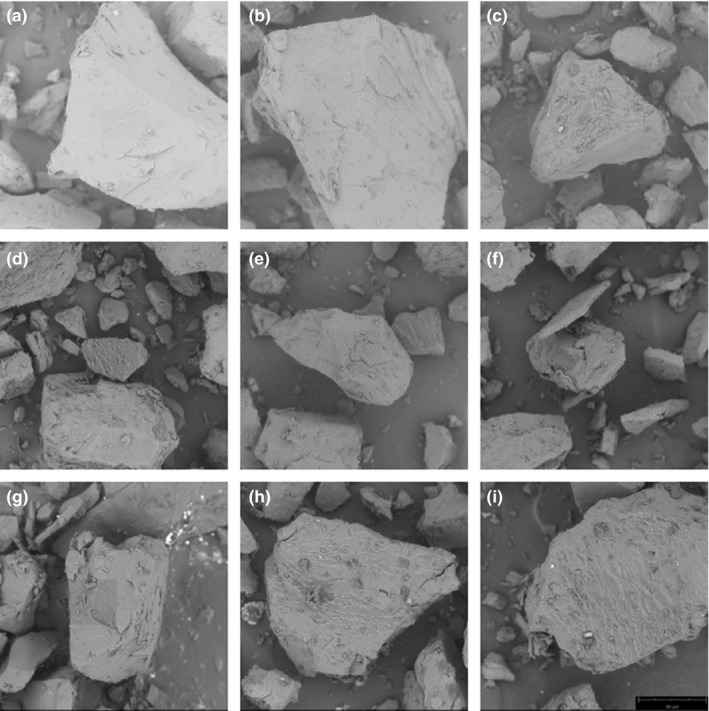
*SEM* of Extruded Corn Flour with Different Cellulose Contents(a, b, c… represent different cellulose additions,a is 0,b is 1,c is 3…and i is 15%)

Microscopic morphology of the extruded samples with different cellulose additions was measured by a scanning electron microscope (“SEM”). Results are shown in Figure 6.

As can be seen from Figure 6, wrinkles on surface of the samples increased as cellulose content increased. As content of cellulose acted as a filler in the melt during the extrusion process increased, a swelling reaction occurred, which affected gelatinization of starch and hindered evaporation of internal water from forming a void in the melting process, thereby affecting degree of expansion. Moreover, shearing force received in the sleeve also caused the material to be completely destroyed, resulting in an increase in wrinkles.

## 4 CONCLUSION

Addition of cellulose cause changes in hardness, puff, color, enthalpy, rheological properties and micromorphology of the product. Increase of dietary fiber in corn flour had an increasing effect on physical and chemical properties of the extruded corn flour. The addition of cellulose can effectively increase the degree of puffing of extruded products. When amount of cellulose added was 15%, hardness was increased by 403.53%, degree of expansion was reduced by 40.24%, L^*^ value was increased by 50.05%, a^*^ value was decreased by 49.63%, and thermal enthalpy of the raw material powder was reduced by 40.57%, the extruded sample paste reduced elasticity and viscosity and exhibited a solid‐like character. Results of micromorphology analysis showed that wrinkles on surface of the extruded samples increased with the increase of cellulose addition.

## CONFLICT OF INTEREST

None.
